# The treatment process of a giant phyllodes tumor of the breast: a case report and review of the literature

**DOI:** 10.3389/fonc.2024.1382985

**Published:** 2024-04-30

**Authors:** Yujun Tong, Siyu Liu, Lijuan Zhao, Zhen Zhang, Haiyan Hu, Yu Jing, Tiantian Liang

**Affiliations:** ^1^ Department of Breast Center, Mianyang Central Hospital, School of Medicine, University of Electronic Science and Technology of China, Mianyang, China; ^2^ Department of Neurology, Mianyang Central Hospital, School of Medicine, University of Electronic Science and Technology of China, Mianyang, China; ^3^ Department of Pharmacy, Mianyang Central Hospital, School of Medicine, University of Electronic Science and Technology of China, Mianyang, China

**Keywords:** giant phyllodes tumor, recurrence, diagnosis, treatment, surgery

## Abstract

Giant phyllodes tumors are rare fibroepithelial tumors that are usually larger than 10 cm in diameter, have rapid tumor growth, and are easily recurrent. They are frequently accompanied by skin necrosis and infection, particularly in malignant phyllodes tumors. This case report presents a 50-year-old woman who presented to the hospital with a huge left breast mass that was ruptured and infected. The patient received anti-infective treatment and underwent mastectomy and skin grafting, which indicated a malignant phyllodes tumor. The tumor was completely excised after a local recurrence in the chest wall 6 months post-surgery. Unfortunately, one year later, the patient pass away due to multiple organ failure. Giant phyllodes tumor management presents challenges to the surgeon. This case is being presented to enhance understanding and treatment of phyllodes tumors, specifically giant malignant phyllodes tumors, with the aim of improving patients’ quality of life.

## 1 Introduction

Phyllodes tumors are an uncommon occurrence, comprising less than 1% of all breast tumors (1). They are biphasic fibroepithelial tumors that encompass both stromal and epithelial components. Typically, these tumors manifest as painless nodules with a propensity for rapid growth within a short duration. The World Health Organization (WHO) has classified phyllodes tumors into three distinct histotypes based on clinicopathological characteristics: benign, borderline, and malignant. These histotypes are observed in approximately 60%-75%, 15%-20%, and 10%-20% of cases, respectively (2). Most phyllodes tumors are around 4 cm in size, but less than 10% grow larger than 10 cm and are called giant tumors (3). Giant malignant phyllodes tumors are rare and hard to treat. Surgery remains the primary treatment option, with very few surgical studies on giant phyllodes tumors. This case involved a very aggressive giant malignant phyllodes tumor that recurred, spread, and died within a short period of time despite undergoing mastectomy.

## 2 Case report

**Figure 1:**
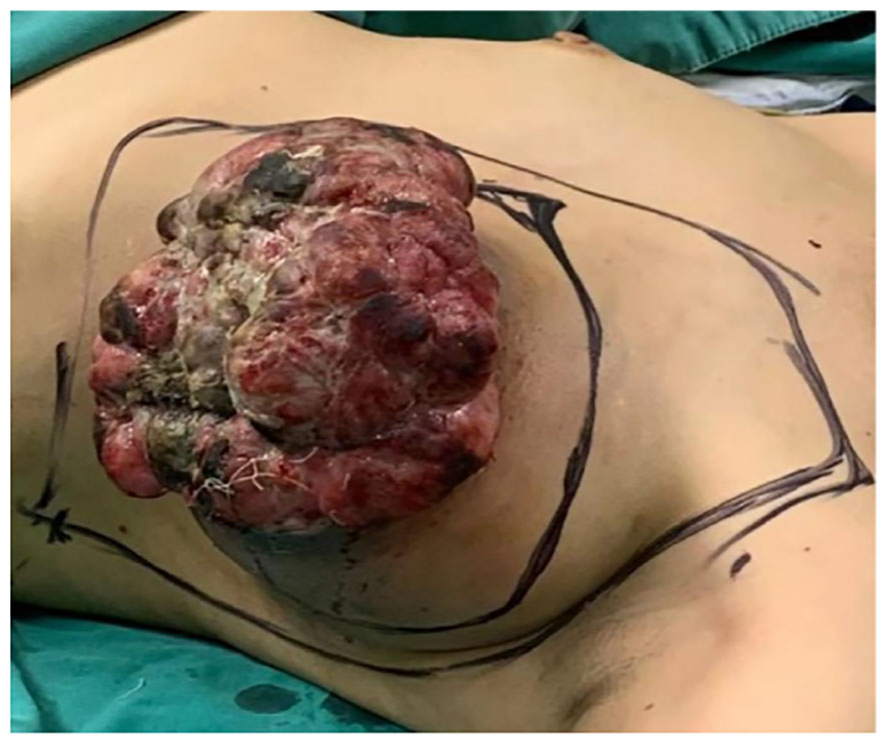
The preoperative measurement of the left breast mass was 17 cm x 15 cm x 10 cm.

**Figure 2:**
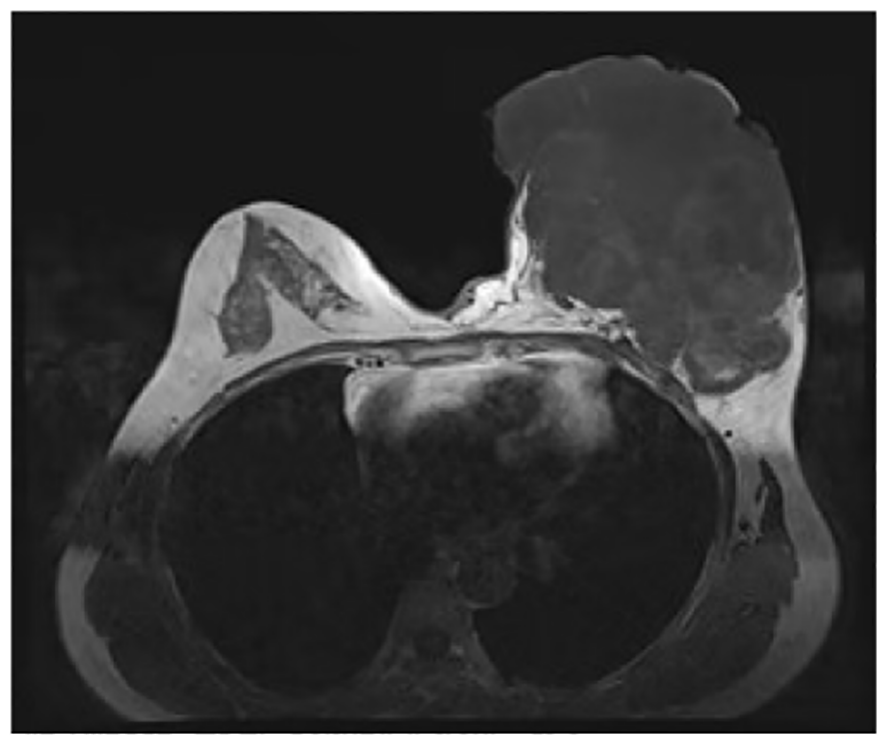
Contrast-enhanced magnetic resonance imaging findings. The boundary between the mass and the pectoralis major muscle was indistinct, with persistent signs of infection observed.

In June 2019, the patient identified a small lump in her left breast without associated discomfort or identifiable etiology, yet she refrained from seeking medical attention or undergoing evaluation. By September 2020, the lump exhibited rapid growth prompting her to present at a clinic where she received a misdiagnosis of mastitis, leading to an incision and drainage procedure. Subsequently, the wound failed to heal, and the tumor extended along the incision site. In November 2020, she was admitted to the Department of Breast Surgery at Mianyang Central Hospital, affiliated with the University of Electronic Science and Technology of China in Mianyang, China. We created a timeline of key points in the patient’s diagnosis and treatment (**Table 1**). Her vital signs were as follows. Physical examination revealed a large, irregular mass in the left breast measuring 17 cm x 15 cm x 10 cm with unclear borders and rupture (**Figure 1**). The skin over the mass became necrotic, with a foul odor and oozing fluids. This caused slight pain. The patient did not have a fever. No enlarged lymph nodes were found in the axilla or clavicle. The patient had no other medical history. Her father died from liver and pancreatic cancer. Lab results revealed an abnormal complete blood count, indicating a high white blood cell count of 10,610/μl and a high platelet count of 468,000/μl. The bacterial culture of exudate was positive for Pseudomonas aeruginosa and Enterococcus faecalith. Liver and kidney function, tumor markers, and other tests were normal. The mass had ruptured upon admission, so mammography and ultrasonography were not possible. Contrast-enhanced magnetic resonance imaging (MRI) showed a large mass in the left breast with an unclear border (**Figure 2**). The imaging studies, including computed tomography scan of the thorax, abdominal ultrasound, and bone scan, did not show any signs of distant metastasis. The results of the core needle biopsy supported the diagnosis of a phyllodes tumor in the breast. The patient received a four-day course of intravenous antibiotics and wound care. The white blood cell count of 7880/μl indicated successful management of the infection before undergoing mastectomy.

**Table 1:** A timeline of key points in the patient’s diagnosis and treatment.

Date	Symptom	Diagnosis	Treatment
June 2019	A painless mass on the left breast was found without any pain or discomfort	No	No treatment was given
September 2020	Rapid growth of the left breast mass	Misdiagnosis of mastitis at a clinic	Incision and drainage procedure performed.
November 2020	Left breast mass measured 17 cm x 15 cm x 10 cm with ill-defined and ruptured borders, skin exhibited necrosis, foul odor, and oozing fluid	Giant malignant phyllodes tumor with infection	Four-day course of intravenous antibiotics, wound care, mastectomy, and skin graft
May 2021	Subcutaneous nodule on the anterior part of the sternal body measuring approximately 3.0 cm x 2.5 cm.	Local recurrence of the malignant phyllodes tumor	Localized expended resection of the mass and displacement of the medial right breast gland to the anterior thoracic region
June 2022	Multiple metastases in the liver, lung, and brain	Patient succumbed to multiple organ failure	–

**Figure 3:**
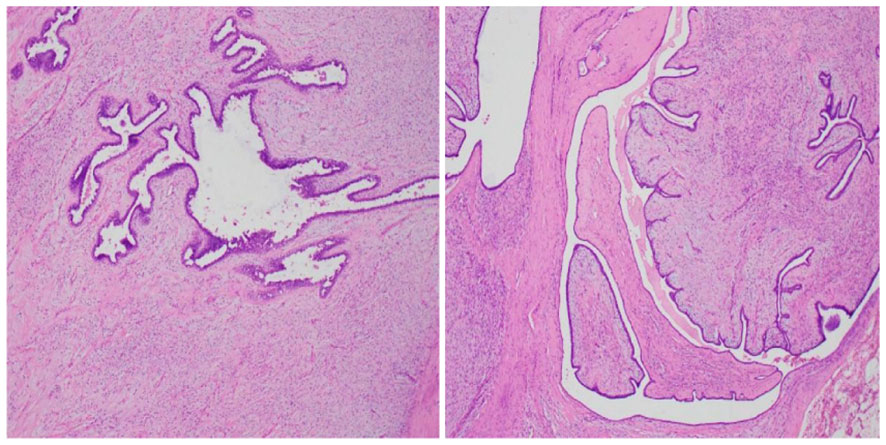
Histological analysis of the surgical specimen demonstrated heightened cellularity surrounding the glandular structures, along with cellular atypia, elevated mitotic activity, as well as hemorrhage and necrosis (HE staining; magnification 100x).

Following standard disinfection and draping procedures, the entirety of the mass was meticulously wrapped and secured using sterile gauze to prevent potential contamination. During the surgery, surgeons noted significant vascularity of the tumor and minor adhesion to the base of the pectoralis major fascia. Subsequently, a horizontal oval incision was made to excise the entire breast, encompassing the nipple, areola, and pectoralis major fascia, while preserving the pectoralis major and pectoralis minor muscles. After excision, the wound underwent meticulous irrigation with warm sterile water to remove any debris or blood. Subsequently, hemostasis was achieved. To facilitate wound healing and chest reconstruction, skin from the abdomen was harvested and transplanted onto the chest. The patient was diagnosed with a malignant phyllodes tumor. Follow-up evaluation confirmed successful removal of breast tissue measuring 20 cm × 17 cm, with no tumor spread to the pectoralis major fascia. The histopathological examination demonstrated typical morphological features of malignant phyllodes tumors, marked stromal cellularity, marked stromal cell anisotropy with a few ductal epithelial component visible in the middle, characteristic leaf-like fronds protruding into cystically dilated fissures, mitotic rate of ≥ 10/10 high power fields, stromal overgrowth, and infiltrative tumor borders (**Figure 3**). The patient experienced no wound-related complications during hospitalization and was discharged on the 13th day after surgery.

**Figure 4:**
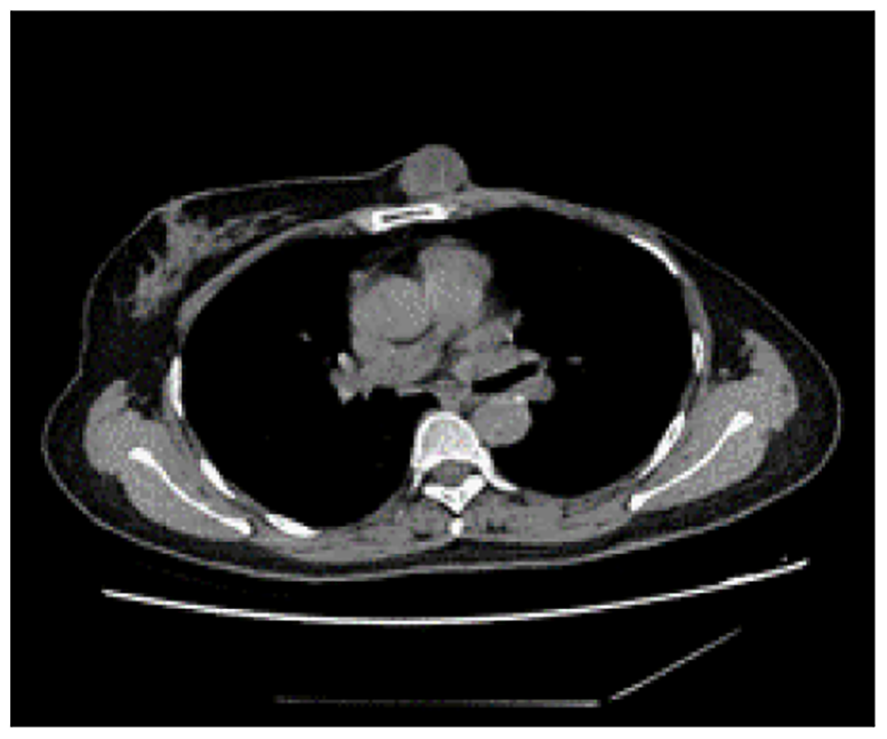
Computed tomography of the thorax identified a subcutaneous mass anterior to the sternum, measuring approximately 3.0 cm x 2.5 cm, characterized by smooth margins and regular morphology.

Although postoperative radiotherapy was recommended due to uncertainty about achieving sufficient negative margins for the ruptured giant tumor, the patient declined. In May 2021, the patient presented to Mianyang Central Hospital with a subcutaneous nodule anterior to the sternal body measuring approximately 3.0 cm x 2.5 cm (**Figure 4**). Subsequently, the patient underwent a localized expended resection of the mass, resulting in a significant defect necessitating an incision in the right inframammary fold to displace the medial right breast gland to the anterior thoracic region, with negative surgical margins. The final histological diagnosis revealed a recurrent malignant phyllodes tumor. Subsequently, the patient declined postoperative radiotherapy despite medical advice. After one year of monitoring, the patient developed liver, lung, and brain metastases, leading to multiple organ failure and death. The reporting of this study follows CARE guidelines (4).

## 3 Discussion

According to the WHO classification, phyllodes tumors are fibroepithelial neoplasms characterized by distinct intracanalicular architectural patterns and leaf-like stromal fronds. These features are accompanied by luminal epithelial and myoepithelial layers and stromal hypercellularity. Phyllodes tumors are stratified into three subgroups (benign, borderline, and malignant) based on criteria including stromal cellularity, stromal atypia, stromal overgrowth, mitotic activity, and tumor margins (as shown in **Table 2**) (2, 5). Immunohistochemical analysis has shown positive expression of vascular endothelial growth factor receptor (VEGFR), P53, CD117, P16, and epidermal growth factor receptor (EGFR) in malignant phyllodes tumors. Studies have demonstrated that malignant phyllodes tumors exhibit lower expression of CD34, with CD34 expression showing an inverse correlation with unfavorable histological characteristics (6–8). The primary objective is to differentiate malignant phyllodes tumors from spindle cell metaplastic breast carcinomas and primary breast sarcomas.

**Table 2:** Histopathological features of benign, borderline and malignant phyllodes tumors of the breast.

Subgroups	Stromal cellularity	Stromal atypia	Stromal overgrowth	Mitosis (/mm^2^ HPFs)	Tumor margin
Benign	Mild	Mild	Absent	< 2.5	Well defined
Borderline	Moderate	Moderate	Absent or focal	2.5 - 5.0	Well defined and focally infiltrative
Malignant	Marked	Marked	Present	> 5.0	Infiltrative

HPE, high-powered field.

Spindle cell metaplastic breast carcinomas may contain varying amounts of malignant epithelial components, with some cases lacking epithelial components entirely or showing heterologous mesenchymal differentiation. The identification of diffuse broad-spectrum cytokeratin or p63 expression in malignant spindle cells may indicate the presence of metaplastic carcinoma, as these proteins are typically not expressed in malignant phyllodes tumors (9, 10). Breast sarcomas are a rare subset of malignant tumors that arise from the mesenchymal tissue of the breast, characterized by a lack of distinctive histological features. In contrast to malignant phyllodes tumors, which typically exhibit a sarcomatous pattern in the stromal tissue and a benign epithelial component, breast sarcomas do not display benign epithelial components or leaf-like stromal fronds (11). Studies have suggested a potential association between genetic predispositions, such as Li-Fraumeni syndrome, and an elevated risk of developing breast sarcomas through TP53 mutations (12, 13).

Diagnosis of breast phyllodes tumors prior to surgical intervention remains challenging. Ultrasound and mammography lack specificity in imaging phyllodes tumors. Prior studies have not established the efficacy of MRI in the detection of breast phyllodes tumors (14). Histopathological assessment remains the gold standard for diagnosing these tumors. Ultrasound-guided core needle biopsy (CNB) has emerged as the preferred method for obtaining tissue samples from suspicious breast lesions for histopathological examination due to its safety, speed, and convenience. Nonetheless, the heterogeneous nature of phyllodes tumors poses a challenge in distinguishing them from epithelial neoplasms or fibroadenomas during biopsy procedures, potentially leading to missed diagnoses. Research indicates that CNB has an approximate 50% accuracy rate in diagnosing phyllodes tumors, with larger tumor size correlating significantly with discordant biopsy results (15).

Surgical intervention is the mainstay of treatment for phyllodes tumors. Benign phyllodes tumors exhibit no heightened risk of local recurrence following any type of resection. Conversely, mastectomy for borderline and malignant phyllodes tumors has been shown to improve local recurrence rates and disease-free survival (16–18). The majority of studies have demonstrated a lack of statistically significant disparity in overall or cancer-specific survival rates among patients with phyllodes tumors who underwent wide excision compared to mastectomy. Margins are still a matter of debate. While systematic reviews have not found a consistent correlation between positive margins and local recurrence in benign and borderline phyllodes tumors (14), a negative margin has been consistently associated with decreased recurrence risk in malignant phyllode tumors (19). Moo et al. discovered that patients with benign phyllodes tumors who underwent re-excision due to positive or close margins did not experience lower rates of local recurrence (20). The National Comprehensive Cancer Network (NCCN) recommends a negative margin of more than 1cm for malignant phyllodes tumors. However, Thind et al. found no significant difference in local recurrence or survival rates between borderline and malignant phyllodes tumors with margins of 1 cm versus >1cm (21). Failure to accurately measure tumor margins during surgery, particularly in cases of giant and ruptured tumors with unclear borders, may contribute to early recurrence of phyllodes tumors. It is increasingly acknowledged that the appropriate surgical margin width should be tailored to the specific subtype of the phyllodes tumor. Mastectomy is primarily employed for larger and recurrent tumors, particularly those of a malignant nature. Giant phyllodes tumors typically encompass the entire breast and present challenges in achieving adequate resection margins. It is important to recognize that in cases where phyllodes tumors reach significant size, complete removal of breast tissue and the tumor-infiltrated soft tissues can decrease the likelihood of local tumor recurrence. Patients with positive surgical margins for benign phyllodes tumors could be follow-up closely, while borderline and malignant phyllodes tumors with margins of more than 1cm should be a localized expanded resection. Khosravi-Shahi, P et al. demonstrated that over 90% of malignant phyllodes tumor cases did not exhibit axillary lymph node metastasis, thus supporting the recommendation to avoid axillary surgery (22).

Certain researchers argue that breast reconstruction may pose a potential risk for phyllodes tumors, which are known for their high recurrence rate. While certain studies suggest that recurrence is not directly linked to breast reconstruction, the possibility of local recurrence could lead to reconstruction failure, necessitating multiple surgeries and imposing additional physical, psychological, and financial strain on patients (23, 24). Therefore, timely surgical intervention is recommended for giant borderline or malignant phyllodes tumors to prevent complications such as tumor compression of the chest wall and invasion of the thoracic cavity. Preoperative assessment of tumor compression or invasion of the chest wall is essential (25). Surgical procedures should be performed cautiously to ensure complete excision of the tumor and to prevent residual disease or compromise of the tumor’s integrity, which may result in short-term recurrence (26). Careful handling of the tumor envelope during surgery is crucial, requiring blunt separation to maintain its integrity. In cases where the envelope is compromised, meticulous identification and dissection of the tumor, along with demarcation of normal tissue, are imperative. Preservation of normal skin and muscle is a priority to adequately cover the wound during complete tumor dissection and attainment of negative margins. For cases where the tumor was ulcerated prior to surgery, it is recommended to meticulously cover and isolate the ulcerated area with gauze during the procedure to prevent contamination of the operative site. Management of large skin defects may necessitate plastic restoration techniques such as skin grafts or rotation flaps.

Adjuvant radiotherapy has been shown to decrease local recurrence rates in borderline and malignant phyllodes tumors, especially following breast-conserving surgery, and is particularly beneficial for individuals under 45 years of age and those with tumors larger than 5cm (27, 28). Nevertheless, there is currently no evidence supporting the efficacy of postoperative adjuvant radiotherapy in improving overall survival for primary phyllodes tumors, as indicated by the NCCN guidelines. The efficacy of adjuvant chemotherapy, endocrine therapy, and targeted therapy for phyllodes tumors remains unproven.

Phyllodes tumors predominantly metastasize hematogenously, with distant metastases occurring in 9% of malignant cases, most commonly in the lungs and bones. Recurrence rates of 10-17%, 14-25%, and 23-30% have been reported in the literature for benign, borderline, and malignant phyllodes tumors, respectively (29). Additionally, the pathology of recurrent tumors often presents a more severe condition. Patients afflicted with malignant phyllode tumors who experience distant metastases face a grim prognosis, with an average survival time of 4 to 17 months (30). Research has not identified a disparity in overall survival rates between mastectomy and locally extended resection following recurrence. Therefore, in accordance with NCCN guidelines, wide margin re-excision and postoperative radiotherapy may be deemed appropriate for cases of local recurrence subsequent to the resection of malignant phyllode tumors.

This particular case study serves to enhance the knowledge of diagnosing and treating malignant phyllodes tumors, as well as examining the surgical techniques used for managing large malignant phyllodes tumors. The preservation of the mammoplasty approach may be considered as a viable option when sufficient margins are achieved. Mastectomy may be considered as a treatment option for giant phyllodes tumors in cases where oncoplastic excision fails to yield satisfactory reconstructive outcomes. Limited research exists on the efficacy of combining mastectomy with breast reconstruction in such cases. The complexity of tumor localization poses challenges for reconstruction, necessitating careful preoperative planning to ensure safe and satisfactory oncoplastic outcomes.

## Data availability statement

The raw data supporting the conclusions of this article will be made available by the authors, without undue reservation.

## Ethics statement

The studies involving humans were approved by The studies involving humans were approved by Ethics Committee of Mianyang Central Hospital (S20230213-01). The studies were conducted in accordance with the local legislation and institutional requirements. The patient's personal information has been deidentified. Prior to the study, patient consent to treatment was obtained. Written informed consent was obtained from the individual(s) for the publication of any potentially identifiable images or data included in this article.

## Author contributions

YT: Conceptualization, Writing – original draft. SL: Conceptualization, Writing – original draft. LZ: Writing – review & editing. ZZ: Writing – review & editing. HH: Conceptualization, Writing – review & editing. YJ: Data curation, Formal analysis, Writing – review & editing. TL: Data curation, Formal analysis, Writing – review & editing.
